# A New Rhynchocephalian from the Late Jurassic of Germany with a Dentition That Is Unique amongst Tetrapods

**DOI:** 10.1371/journal.pone.0046839

**Published:** 2012-10-31

**Authors:** Oliver W. M. Rauhut, Alexander M. Heyng, Adriana López-Arbarello, Andreas Hecker

**Affiliations:** 1 Bayerische Staatssammlung für Paläontologie und Geologie and Department of Earth and Environmental Sciences, Ludwig Maximilians University, Munich, Germany; 2 GeoBioCenter, Ludwig Maximilians University, Munich, Germany; 3 Max-Planck-Gesellschaft, Generalverwaltung, Munich, Germany; Raymond M. Alf Museum of Paleontology, United States of America

## Abstract

**Background:**

Rhynchocephalians, the sister group of squamates (lizards and snakes), are only represented by the single genus *Sphenodon* today. This taxon is often considered to represent a very conservative lineage. However, rhynchocephalians were common during the late Triassic to latest Jurassic periods, but rapidly declined afterwards, which is generally attributed to their supposedly adaptive inferiority to squamates and/or Mesozoic mammals, which radiated at that time. New finds of Mesozoic rhynchocephalians can thus provide important new information on the evolutionary history of the group.

**Principle Findings:**

A new fossil relative of *Sphenodon* from the latest Jurassic of southern Germany, *Oenosaurus muehlheimensis* gen. et sp. nov., presents a dentition that is unique amongst tetrapods. The dentition of this taxon consists of massive, continuously growing tooth plates, probably indicating a crushing dentition, thus representing a previously unknown trophic adaptation in rhynchocephalians.

**Conclusions/Significance:**

The evolution of the extraordinary dentition of *Oenosaurus* from the already highly specialized Zahnanlage generally present in derived rhynchocephalians demonstrates an unexpected evolutionary plasticity of these animals. Together with other lines of evidence, this seriously casts doubts on the assumption that rhynchocephalians are a conservative and adaptively inferior lineage. Furthermore, the new taxon underlines the high morphological and ecological diversity of rhynchocephalians in the latest Jurassic of Europe, just before the decline of this lineage on this continent. Thus, selection pressure by radiating squamates or Mesozoic mammals alone might not be sufficient to explain the demise of the clade in the Late Mesozoic, and climate change in the course of the fragmentation of the supercontinent of Pangaea might have played a major role.

## Introduction

The recent genus *Sphenodon* is the only living representative of the Rhynchocephalia, the sister taxon of the Squamata (lizards and snakes) within the Lepidosauria [1]. Due to the long history of the group, reaching back to at least the Middle Triassic (∼235 million years), and the supposedly primitive morphology of the genus, *Sphenodon* is often regarded as a “living fossil”. Thus, this taxon is frequently used as a model organism for a basal diapsid in investigations dealing with the evolution of reptiles, even in recent studies ranging from the structure of the pineal organ [2] to tetrapod locomotion [3].

Rhynchocephalians were a common component of Mesozoic small vertebrate faunas [4], [5], and research in the past thirty years has shown that they were not only taxonomically and ecologically diverse during this time, but also showed a remarkable morphological variability [6]. Indeed, several of the features that were thought to represent the plesiomorphic condition in *Sphenodon*, such as the closed lower temporal arcade, were recently demonstrated to be apomorphic reversals to a condition resembling the ancestral morphology instead (e.g. [7], [8]). Nevertheless, all rhynchocephalians but the most basal forms are characterized by a very specialized dentition pattern, in which the teeth are fully acrodont, no tooth replacement is present in post-hatchling individuals, and additional teeth are added at the posterior end of the tooth row during ontogeny [9]. Although tooth shape itself is quite variable in rhynchocephalians [10], this special type of tooth development was thought to limit their adaptive potential [11]. Consequently, the demise of the group in the later Mesozoic has been linked to the adaptive radiation of squamates and/or mammals (see [5]).

**Figure 1:**
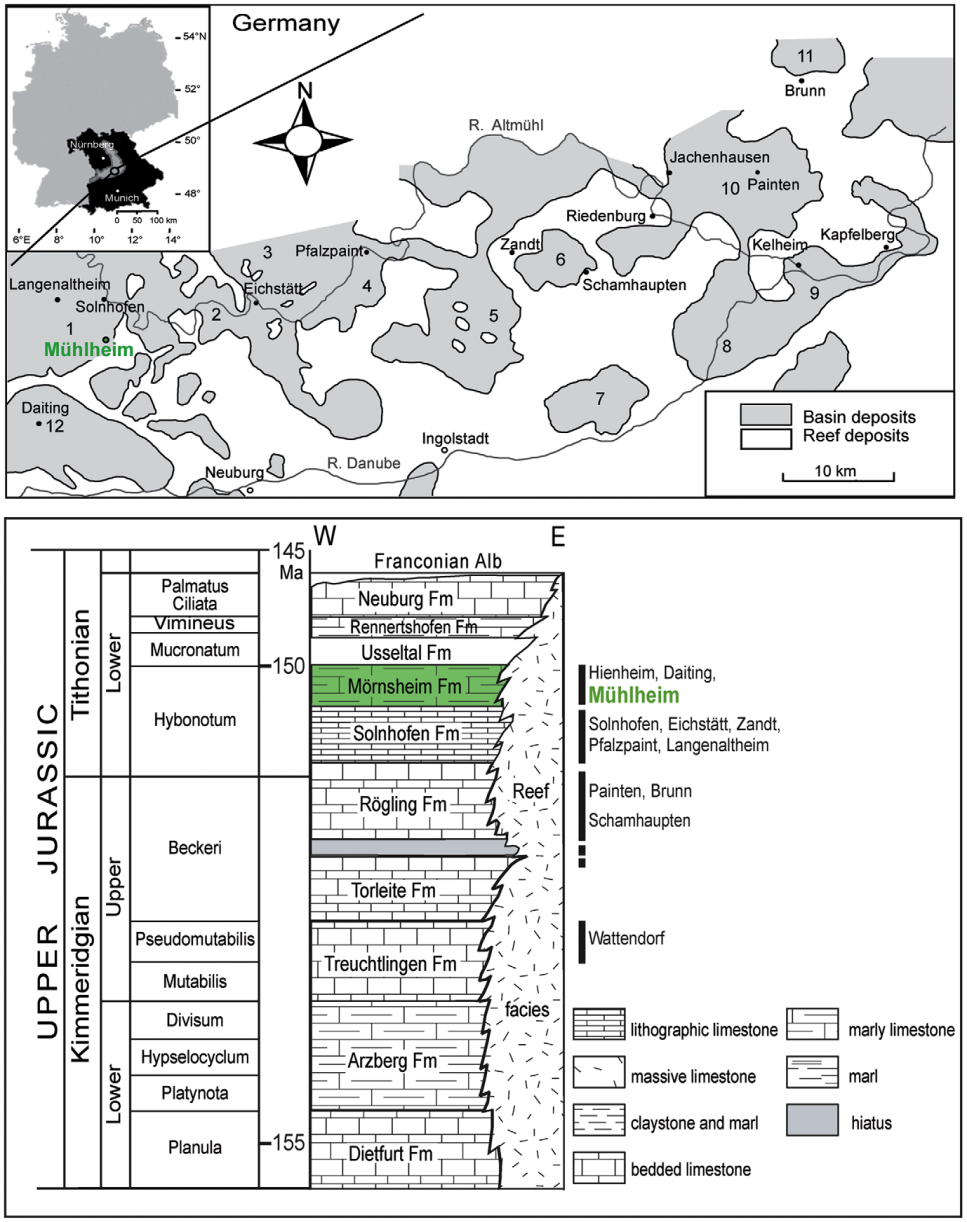
Geographical and stratigraphic setting of the locality of the new taxon. Modified from [16], [54], [55].

Here we report on a new taxon of rhynchocephalian from the Late Jurassic of southern Germany that further underlines the morphological and ecological diversity of rhynchocephalians and indicates high adaptive plasticity even in the dentitions of these animals. The specimen was found in the middle parts of the Moernsheim Formation exposed at Mühlheim (Fig. 1), in a section of platy, siliceous limestones overlying lime bank B-H-5 (“Krebs-Bank”). The marine Tithonian (Upper Jurassic) Moernsheim Formation covers the famous Solnhofen lithographic limestones in the western region of the Franconian Alb in northern Bavaria. In contrast to the underlying Solnhofen Formation, the sediments of the Moernsheim Formation are more heterogeneous, consisting of mainly siliceous limestones in alternate bedding with marly and limey sections, and with intercalated banks particularly in the lower parts of the formation.

## Methods

The data matrix was analysed with PAUP 4.0b10 (Swofford 2003), using a branch and bound search. The initial analyses resulted in 119 trees with a length of 187 steps (CI 0.465, RI 0.649, RC 0.302). The strict consensus tree of these trees showed poor resolution, with Oenosaurus representing a member of a polytomy of rhynchocephalians more derived than Planocephalosaurus (Information S1); only pleurosaurs and eilenodontines showed up as monophyletic clades within this polytomy. However, reduced consensus trees of these 119 trees recovered a monophyletic Sphenodontinae, including Oenosaurus, but without further resolution within this clade (Information S1).

The taxon Pamizinsaurus from the Early Cretaceous of Mexico (Reynoso 1997) was identified as a wildcard taxon that strongly influenced the results. This may partially be due to high amounts of missing data (64%) in this taxon; however, that several other taxa have even higher amounts of missing data (Eilenodon 68%; Cynosphenodon: 73%; Toxolophosaurus: 79%) but proved to be less problematic, indicates that the wildcard status of Pamizinsaurus might also be due to some character conflict.

In a next step, we thus removed Pamizinsaurus from the matrix and ran a second analysis with the same settings as above. The analysis resulted in only two most parsimonious trees with a length of 184 steps (CI 0.473, RI 0.654, RC 0.309). The strict consensus tree of these two trees (Fig. 3 and Information S1) is considerably better resolved than that resulting from the full analysis, but, importantly, it does not contradict the results of the latter, neither in the strict consensus, nor in any reduced consensus tree. Thus, we chose these results to illustrate and further explore the relationships of Oenosaurus. Bootstrap (10000 replicates) and Bremer support analyses were carried out on the pruned matrix, and the following list of synapomorphies is based on the strict consensus tree resulting from the pruned analysis.

### Nomenclatorial Acts

The electronic edition of this article conforms to the requirements of the amended International Code of Zoological Nomenclature, and hence the new names contained herein are available under that Code from the electronic edition of this article. This published work and the nomenclatural acts it contains have been registered in ZooBank, the online registration system for the ICZN. The ZooBank LSIDs (Life Science Identifiers) can be resolved and the associated information viewed through any standard web browser by appending the LSID to the prefix “http://zoobank.org/”. The LSID for this publication is: urn:lsid:zoobank.org:pub: 4A00D064-A136-4944-AF8E-427B941FB38C. The electronic edition of this work was published in a journal with an ISSN, and has been archived and is available from the following digital repositories: PubMed Central, LOCKSS.

## Results

### Systematic Palaeontology

Lepidosauria Haeckel, 1866 [12]

Rhynchocephalia Günther, 1867 [13]

Sphenodontia Williston, 1925 [14]

Sphenodontidae Cope, 1871 [15]

*Oenosaurus* gen.nov.

urn:lsid:zoobank.org:act:48C1BA32-BC6D-407D-81E0-7ECFF22D77A3

*Oenosaurus muehlheimensis* sp. nov.

urn:lsid:zoobank.org:act:6648C44F-FDC0-48B2-A6A9-399B17E07D38

#### Etymology

*Oenos*, [oinos] Greek, wine, referring to the Franconian Alb, a famous wine area, where the specimen was found, and *saurus*, [sauros] Greek, lizard. Species name refers to the village of Mühlheim, close to Mörnsheim, at the rim of the valley of the Altmühl in Bavaria.

#### Holotype

**Figure 2:**
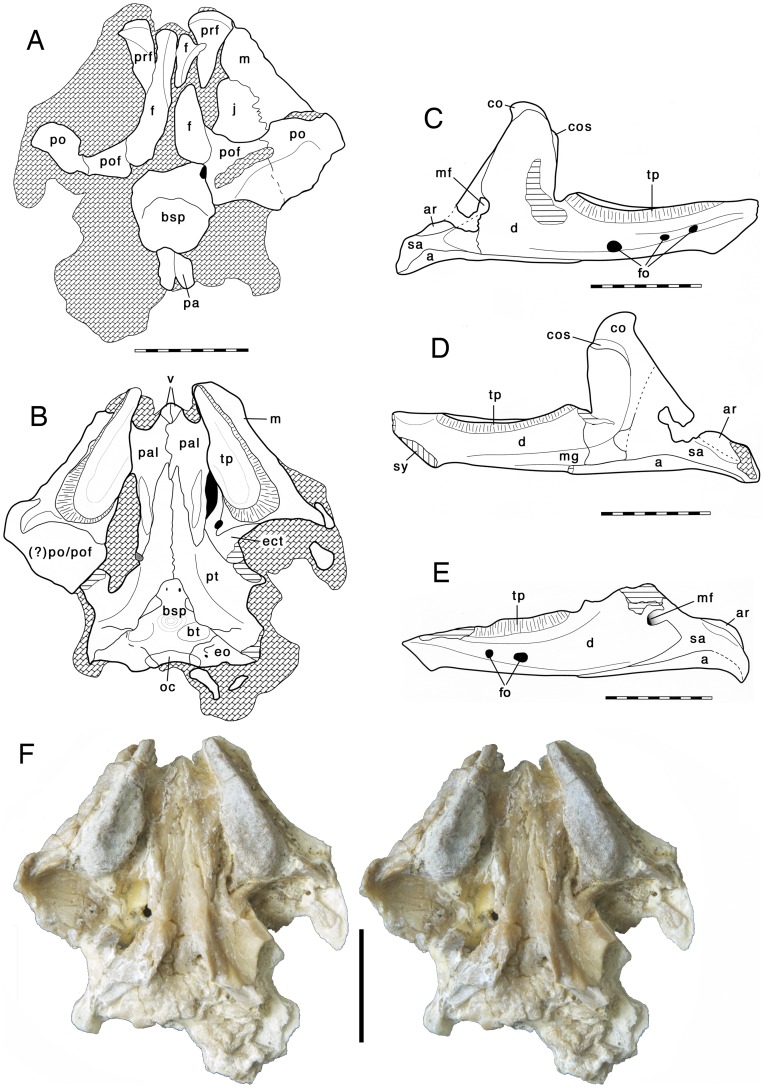
New Late Jurassic sphenodontine rhynchocephalian *Oenosaurus muehlheimensis* (Holotype, BSPG 2009 I 23). (A)–Skull in dorsal view. (B)–Skull in ventral view. (C–D)–Right mandible in lateral (C) and medial (D) views. (E)–Left mandible in lateral view. (F)–Stereophotographs of the skull in ventral view. Abbreviations: a, angular; ar, articular; bsp, basisphenoid; bt, basal tubera; co, coronoid; cos, coronoid shelf; d, dentary; ect, ectopterygoid; eo, exoccipital/opisthotic; f, frontal; fo, foramen; j, jugal; m, maxilla; mf, mandibular foramen; mg, Meckelian groove; oc, occipital condyle; pa, parietal; pal, palatine; po, postorbital; pof, postfrontal; prf, prefrontal; pt, pterygoid; sa, surangular; sy, symphysis; tp, tooth plate. Scale bars are 10 mm.

**Figure 3:**
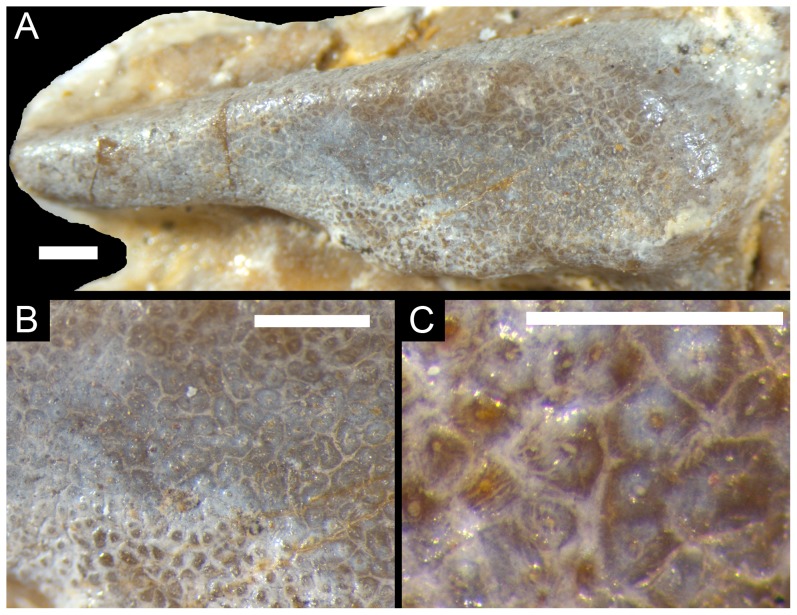
Details of dentition of *O. muehlheimensis* (Holotype, BSPG 2009 I 23). (A)–Left maxillary tooth plate in ventral view (anterior is to the left). (B)–Enlargement of posterior part of left maxillary tooth plate, showing compound structure of the plate. (C)–Further enlargement of left maxillary tooth plate, showing closely packed, worn teeth with central pulp cavities. Scale bars are 1 mm (A, B) and 0.5 mm (C).

**Figure 4:**
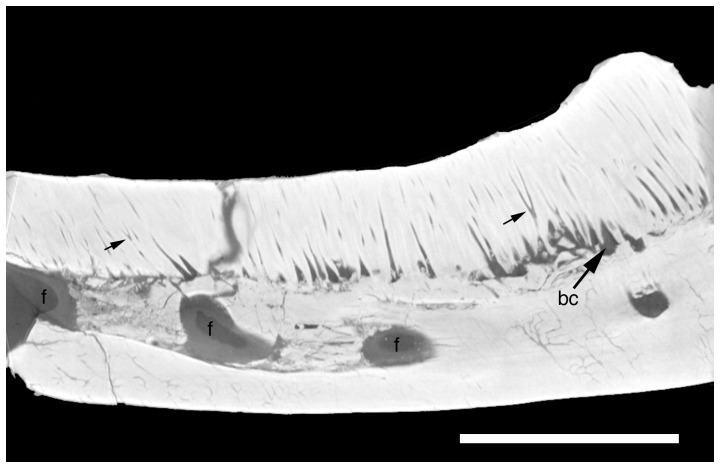
Parasagittal micro CT section of the right dentary of *Oenosaurus muelheimensis*. Posterior is to the right. Abbreviations: bc, basal cavity; f, foramen. Small arrows point to bifurcating dentine channels. Scale bar is 5 mm.

Bayerische Staatssammlung für Paläontologie und Geologie, Munich, Germany, BSPG 2009 I 23; partial skull and complete mandibles (Figs. 2, 3, 4).

#### Locality and horizon

Mörnsheim Formation (Lower Tithonian) [16], “Krautworst Naturstein” quarry in Mühlheim near Mörnsheim, central Bavaria, Germany (Fig. 1).

#### Diagnosis

Small rhynchocephalian with the following autapomorphic characters: maxilla with a medial process at the posterior end; ectopterygoid with a secondary lateral process that contacts the medial side of the maxilla; palatines with a midline contact in ventral view; strongly pronounced lateral longitudinal groove on the dentaries, housing several large foramina; very high coronoid process, the anterior border of which forms an angle of approximately 90° with the tooth row; coronoid with a pronounced shoulder medially; dentition composed of extensive tooth plates formed by a multitude of fused, small, pencil-like teeth.

#### Description

The skull of *Oenosaurus* was preserved in ventral view, with the mandibles slightly displaced from the upper jaws. The skull is diagenetically dorsoventrally compressed and much of the dorsal skull roof is missing, as is the roof of the braincase. However, enough is preserved to record the general shape of the skull and the position and size of the orbitae (Fig. 2a).

The skull seems to have been rather robust. As preserved, it is 24.3 mm long. Unfortunately, the exact skull length cannot be determined, since the premaxillae are missing, but these bones probably did not account for more than an additional 2–3 mm. Thus, the skull was slightly broader (28 mm) than long, as in many rhynchocephalians, but unlike the slender, elongate skull of the marine pleurosaurs [6], [17]. The skull is triangular in dorsal or ventral view (Fig. 2a, b, f). The orbitae are large and placed anteriorly over the maxillary dentition, as in *Sphenodon* and *Clevosaurus* [6]. Premaxillae and nasals are missing. The frontals are elongate and narrow, and flanked by the prefrontals anterolaterally (Fig. 2a). There is no trace of a lacrimal, though it cannot be ruled out completely that this might be due to preservation. The posterior part of the skull roof is poorly preserved and nothing can be said about the shape and size of the temporal fenestrae. A small fragment of the parietals shows that these bones were fused and formed a narrow sagittal crest medially. The postfrontals and postorbitals formed a broad shelf anterior to the dorsal temporal fenestra. The jugal was broad and reached anteriorly to almost half-length of the orbit. The maxillae are massive and broad, but rapidly narrow anteriorly. Posteriorly, the maxilla has a stout lateral process for the contact with the jugal and a smaller medial process that slots into the forked lateral process of the ectopterygoid.

The palate is only missing most of the vomer and the posterior ends of the quadrate wings of the pterygoids (Fig. 2b, f). The vomers were small. Their posterior ends separate the anterior ends of the palatines, but do not reach the pterygoids posteriorly, so that the palatines meet at the midline. Such a midline contact of the palatines was illustrated for the marine pleurosaurs by Carroll and Wild [17], but not described in the text, and the line of contact is dotted in their reconstruction, so that some uncertainty of the condition in these animals remains. The palatines contact the maxillae laterally in a broad suture at about one third of the length of the latter bone, and form the posterior border of the internal choanae. The bones flank the pterygoids laterally over most of the length of the anterior process of the latter bone, taper posteriorly, and end a short way posterior to the posterior end of the maxillae. As in all sphenodontids, there is a raised ridge along the lateral edge of the posterior half of the palatines, but, due to preservation, it is uncertain if this ridge bore teeth, as it is the case in most taxa [7], [8]. In contrast with derived rhynchocephalians [18], [19], but like the situation in basal forms [7], [20], the ridge diverges from the maxillary posteriorly and is more or less parallel to the midline of the skull. The pterygoids taper anteriorly and broaden abruptly posteriorly towards the ventrolaterally extending pterygoid wings. There is no interpterygoid vacuity, but the pterygoids form a midline suture up to the basicranial articulation. Posteriorly, the slender, but well-developed, quadrate wings of the pterygoids diverge away from the ventral side of the braincase. A small fragment of bone lateral to the remnants of the left quadrate wing of the pterygoid might represent a remain of the pterygoid wing of the quadrate. Unlike basal rhynchocephalians, there are no teeth on the pterygoid. No suture between the quadrate wing of the pterygoid and the pterygoid wing of the quadrate is visible in the preserved portions, so the latter bone, which is not preserved, might have had a rather short pterygoid wing.

The ectopterygoid attaches anterolaterally to the pterygoid wing. It is unusual in that it has two lateral processes, one that contacts the posterior end of the maxilla, as in other rhynchocephalians, and a more anterior one that extends anterolaterally lateral to the palatine and contacts the medial side of the maxilla (Fig. 2b, f). This process forms the posterior border of the suborbital fenestra, which is surrounded by the palatine, maxilla, and ectopterygoid. Thus, the pterygoid is excluded from the rim of this fenestra, as in many other sphenodontids, but not more basal rhynchocephalians. In contrast to clevosaurs [21], [22], [23] there is no contact between the palatine and the ectopterygoid lateral to the fenestra. Together with the medial process of the maxilla, the secondary anterolateral process frames a large foramen lateral to the palatine ridge.

Only the floor of the braincase and the ventral parts of the occiput are preserved. The basisphenoid is narrow at the basicranial articulation, but rapidly widens posteriorly. The basipterygoid processes seem to be stout and short, but are mostly hidden by the pterygoids. The basisphenoid has a broad, shallow depression on the ventral side between the basipterygoid processes and the low, widely separated basal tubera. The occipital condyle is broad and not separated from the basioccipital body by a constricted neck, similar to the situation in the modern *Sphenodon*.

The lower jaw is 33 mm long and is especially notable for its high coronoid process, which exceeds the tooth row by approximately 8.7 mm in the right mandible and is thus almost 1.5 times higher than the body of the dentary (Fig. 2c, d). The dentary accounts for most of the length of the mandible, and, as in all rhynchocephalians, a posteroventral process of the dentary reaches posteriorly beyond the coronoid process to the level of the anterior margin of the mandibular articulation. A large incision in the posterodorsal margin of the dentary marks the enlarged mandibular foramen (Fig. 2c, e). A pronounced groove with several large foramina is present on the lateral side of the dentary, and its dorsal margin seems to be formed by a secondary bone skirt [5], [23], which probably accounts at least partially for the strong transverse thickening of the tooth-bearing part of the dentary. Anteriorly, the ventral margin of the bone extends ventrally to form a small, triangular “chin”, as it is present in many rhynchocephalians (e.g. [20], [24]–[26]). There is no splenial.

The postdentary bones are entirely restricted to the posterior half of the mandible. The surangular makes up the posterior margin of the ventral part of the coronoid process and continues posteriorly to the end of the mandible. The mandibular articulation is developed as a longitudinal dorsal ridge on the articular. The ridge is sharp-edged dorsally and slightly displaced to the lateral side. Medially, it is flanked by a slightly anteroposteriorly concave medial bulge that becomes more pronounced posteriorly. This morphology is similar to the condition in derived rhynchocephalians, possibly indicating a propalinal movement of the lower jaw. The retroarticular process is short and stout. The medial side of the coronoid is thickened and forms a pronounced shelf below the tip of the coronoid process.

The most unusual character of the new taxon is the dentition. Unlike the situation in any other rhynchocephalian, broad tooth plates are present in both the maxillaries and dentaries. The maxillary tooth plates (Fig. 2b, f, 3a) are broad posteriorly and narrow in their anterior half. They are 13 mm long and 4 mm wide at their widest part. The lateral edge of the tooth plates is slightly raised and shows a few small bumps. The dentary tooth plates are elongate and oval to subrectangular in outline, with a length of 13 mm and a maximum width of 3.5 mm. CT data shows that the central part of the tooth plate is placed in a broad depression on the dorsal surface of the mandible, though the tooth implantation can still be regarded as acrodont rather than protothecodont, as it is the case in some basal rhynchocephalians [27].

Under closer inspection, the surfaces of the tooth plates show small, but clearly defined, round, oval, or angular subunits, made up of concentric dentine layers with a small central cavity (Fig. 3). Fine striations radiate outwards from the central cavity. Micro CT-images of the dentary tooth plate show that these dentine tubes extend over the entire height of the plate (Fig. 4). In the basal part, the central cavities become larger and form large, elongate cavities at the base of the tooth plate. In some instances, the central cavities of the dentine tubes bifurcate in their course from the base to the occlusal surface. Tooth enamel seems to be absent, but the dentine tubes are tightly cemented together towards the occlusal surface. At the occlusal surface, the central cavities of the tubes also seem to be filled with cement. The dentine tubes are generally somewhat larger in the central parts of the tooth plate, but become smaller towards the margins (Fig. 3a). The dentine tubes reach further ventrally in the anterior part of the dentary than in the posterior part, which might indicate a later addition of posterior dentine tubes during ontogeny, as it is the case with individual teeth in other rhynchocephalians [9].

### Systematic position

**Figure 5:**
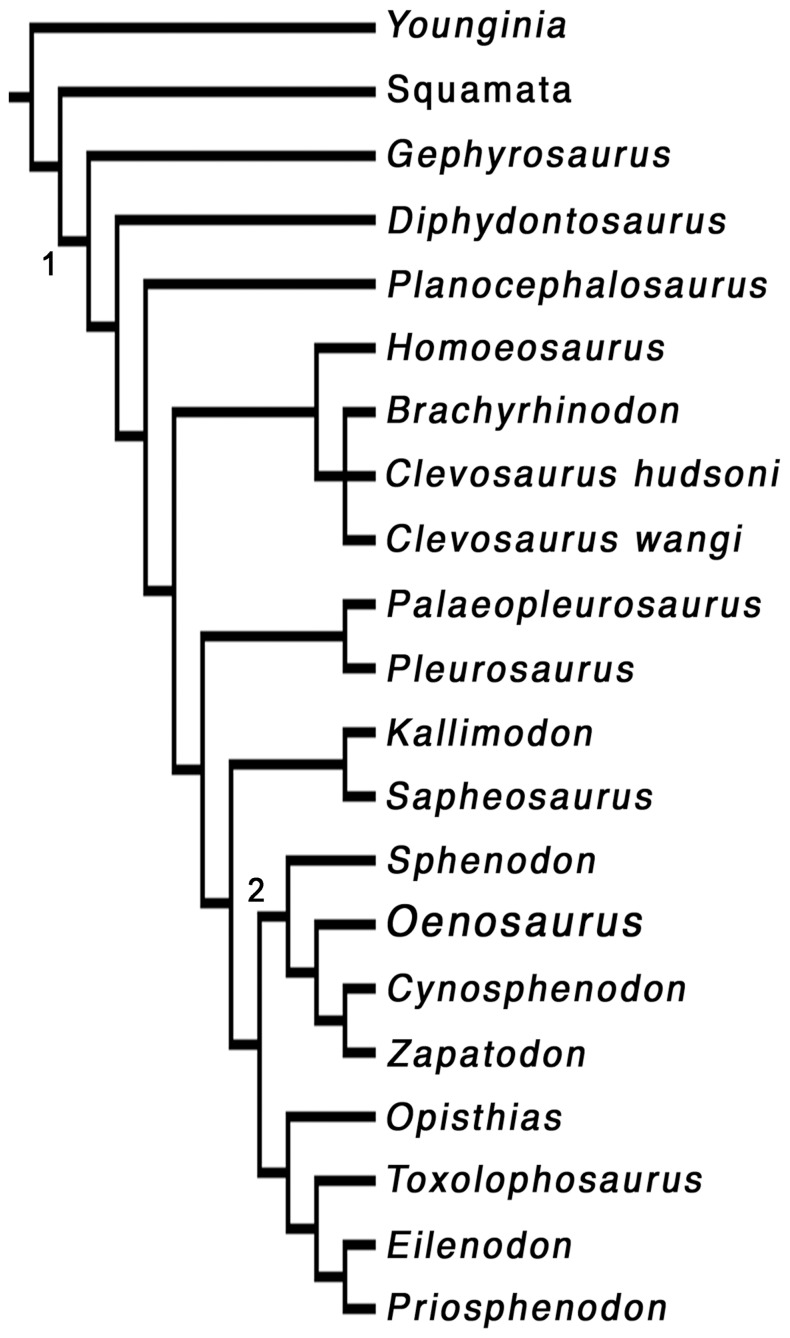
Phylogenetic relationships of *Oenosaurus muehlheimensis* within rhynchocephalians. Cladistic analysis of 70 osteological characters in 19 rhynchocephalian and two outgroup taxa resulted in the recovery of 2 trees with 184 steps (see Information S1). A strict consensus tree shows *Oenosaurus* well nested within sphenodontine rhynchocephalians, as a close relative to the Recent *Sphenodon*. 1, Rhynchocephalia; 2, Sphenodontinae.

Despite its apomorphic cranial morphology and highly unusual dentition, cladistic analysis (see Information S1) places *Oenosaurus* well within the Rhynchocephalia (Fig. 5), because the taxon shows numerous synapomorphies of this clade and more exclusive ingroups, such s Sphenodontia and Sphenodontidae. These characters include a pronounced ridge or tooth row laterally on the palatine, a high coronoid process in the mandible, a posterior process of the dentary that extends posterior to the coronoid process, an enlarged mandibular foramen, the lack of a splenial, and an acrodont dentition [18], [22], [26], [28]–[30]. Within rhynchocephalians, *Oenosaurus* even falls within the clade that includes the Recent form, the Sphenodontinae [5], [24], though support for this placement is weak (see Information S1), and further material of this taxon (and others, which, with the exception of *Sphenodon*, are generally poorly known [5]) might lead to changes in this part of the tree. Nevertheless, the phylogenetic position of the new taxon within Sphenodontidae (sensu [24]) is well supported.

## Discussion

The dentition of *Oenosaurus* is unique amongst tetrapods. Although tooth batteries are known in a small number of groups, including captorhinomorphs, rhynchosaurs, and advanced ornithischian dinosaurs, and plate-like teeth are present in placodonts, the detailed structure of these dentitions was markedly different (see [31]–[33]). The tooth plates of *Oenosaurus* consist of a multitude of minute, closely packed and co-cemented dentine tubes without any discernible pattern of tooth rows or tooth generations. Although the dentine tubes as visible on the occlusal surfaces resemble individual teeth, CT images of the tooth plate show that they are elongate tubes extending for the entire height of the tooth plate, becoming wider towards a basal cavity just above the mandibular bone (Fig. 4). A very similar structure of pulpal canals (sometimes called denteons, due to their similarity to osteones in bone) surrounded by circumpulpal dentine is present in the continuously growing tooth plates of chimaerans and dipnoans (e.g. [34]–[37]). These kinds of tissues are called pleromin in chimaerans and petrodentine in dipnoans and are structurally very similar, though differ in mineralogical composition [38]. Osteodentine (dentine with osteone-like denteones and circumpulpal dentine) is also present in the continuously growing teeth of xenarthran mammals [39]. The structure of the teeth in *Oenosaurus* is similar to that in these taxa, indicating that the tooth plates in this taxon might also have been growing continuously, balanced by wear on the surface of the plate, as in chimaerans [36]. A similar pattern of continuous growth and wear of the teeth was also hypothesized for the Early Cretaceous rhynchocephalian *Ankylosphenodon* by Reynoso [40]. However, the teeth of the latter taxon differ from the tooth plates of *Oenosaurus* in representing separate teeth, being inserted into a deep groove in the jaw, the crowns having a triangular morphology in lateral view, and the presence of enamel at least in unworn teeth.

An interesting problem is the evolution of these tooth plates. The phylogenetic position of *Oenosaurus* shows that this taxon is well nested within taxa that have the specialized acrodont dentition typical for sphenodontids. The very peculiar plates are therefore derived from such a dentition and demonstrate a surprising evolutionary plasticity of such a seemingly highly specialized tooth anlage [9]. A possible, but currently untestable, hypothesis of the evolution of these tooth plates might be that they formed by fusion and modification of the hatchling dentition, which consists of small, peg-like teeth with active tooth replacement in several rhynchocephalians [41], including the modern *Sphenodon* [42], [43]. However, further studies and, possibly, further discoveries of this or other taxa with similar dentitions are necessary to test this hypothesis.

One important question concerns the feeding ecology of *Oenosaurus*. The broad occlusal surface of both the upper and lower jaw argues against a cutting or tearing function, as it is present in most rhynchocephalians [10]. A herbivorous diet, as it has been proposed for the continuously growing dentition of *Ankylosphenodon* [40], also seems unlikely. Although a broad occlusal surface with the possibility of some propalinal movement might be suitable for at least some kind of oral processing in a herbivore, the lack of enamel and thus cutting edges in the teeth and the lack of cropping teeth or a cutting edge in at least the anterior end of the dentary argues against such an interpretation. Furthermore, although a strict relationship between large body size and herbivory in lizards [44] could not be confirmed [45] there seems to be a general relationship between herbivory and large body size in this clade [46]. Thus, the small body size of *Oenosaurus* is more consistent with a carnivorous or omnivorous diet [46]. On the other hand, a crushing dentition in the lizard *Dracaena* was found to be associated with a reinforcement of the palate [47], as it is also the case in the new taxon. The closed interpterygoid vacuity and the bifurcated lateral process of the ectopterygoid, leading to an interlocking articulation between this bone and the maxilla, both help to strengthen this structure and indicate a necessity to withstand high bite forces. Thus, a durophagous diet for *Oenosaurus* seems to be most likely. Unfortunately, since only the skull of this taxon is preserved, nothing can be said about whether it was terrestrial or aquatic. In an aquatic taxon, a diet of molluscs and crabs might be inferred, whereas a terrestrial animal might have fed on snails and insects with a thick endoskeleton. Phylogenetically, the new taxon is nested within fully terrestrial forms, but more complete material is needed to evaluate its ecological preferences.

Despite their specialized dentition, rhynchocephalians are quite variable in their tooth morphology. Rhynchocephalian teeth can be referred to three basic functional types [10]: basal forms usually have small, conical teeth that are mainly used for piercing, most sphenodontids have anteroposteriorly elongate teeth that are used for cutting and slicing, and opisthodontians [18] have transversely expanded teeth capable of grinding and shredding. These different tooth types can roughly be equated with insectivory, generalized carnivory (or piscivory, in the marine pleurosaurs), and herbivory, respectively [10]. The tooth plates of *Oenosaurus* now demonstrate a further type of dentition in rhynchocephalians, a crushing dentition, indicating a durophagous diet. Therefore, the new taxon adds a further trophic adaptation and a previously unknown ecotype to the already recognized ecological diversity of Mesozoic rhynchocephalians [10].

**Figure 6:**
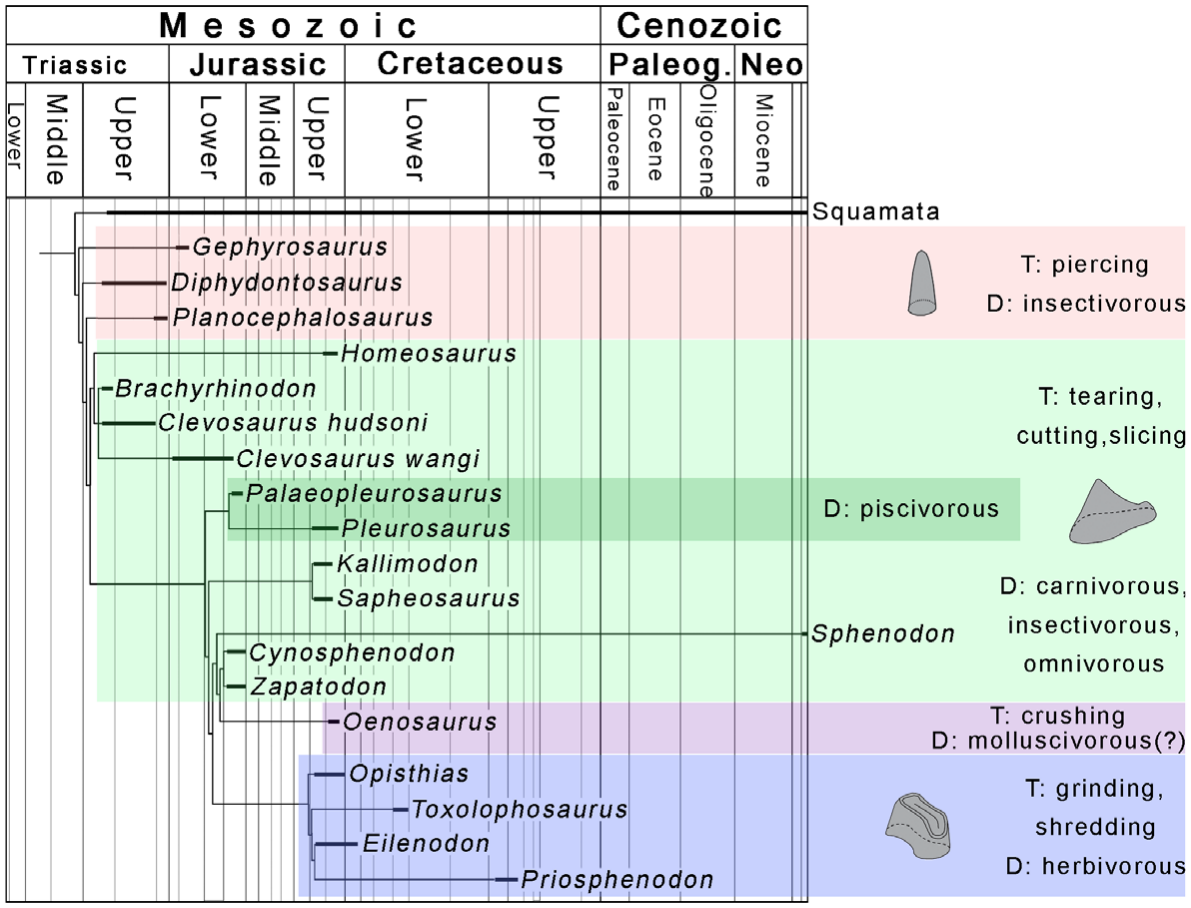
Calibrated phylogeny of rhynchocephalians, indicating different tooth types and dietary interpretations. T, functional tooth type; D, interpretation of dietary preferences.

The occurrence of a highly specialized rhynchocephalian close to the decline of the group in the Cretaceous [4], [48] underlines their morphological and ecological diversity at that time (Fig. 6). In addition to the durophagous *Oenosaurus*, Late Jurassic and Early Cretaceous rhynchocephalians include long-legged, carnivorous or insectivorous terrestrial forms, such as *Homeosaurus*, herbivorous forms with a grinding dentition, such as *Eilenodon*, the enigmatic, probably edentulous aquatic *Sapheosaurus*, and the aquatic, probably piscivorous pleurosaurids [4], [6], [30], [49]. Thus, rhynchocephalians were at their highest morphological and ecological diversity at a time when squamate radiations were already well under way [8] and Mesozoic mammals had achieved a remarkable diversity [50]. This casts doubts on the idea that rhynchocephalians were evolutionary inferior to and displaced by squamates and/or Mesozoic mammals during the later Mesozoic [1], [4], [51]. It has been suggested that rhynchocephalians show a preference for xenic environments in the Mesozoic [23], [52]. Thus, climate change from predominantly xenic conditions in the Jurassic to more mesic conditions in the course of the break-up of Pangaea in the Cretaceous [53], might have been responsible for the decline of this group.

## Supporting Information

Information S1 **Character list, data matrix, analytical procedures, consensus trees, and list of synapomorphies at internal nodes for the cladistic analysis mentioned in the text.**
(DOCX)
