# Supplementary material for: A New Rhynchocephalian from the Late Jurassic of Germany with a Dentition That Is Unique amongst Tetrapods
Source: PLoS One. 2012 Oct 31;7(10):e46839. doi: 10.1371/journal.pone.0046839 (PMC3485277; doi:10.1371/journal.pone.0046839)
Supplement: Information S1 — Character list, data matrix, analytical procedures, consensus trees, and list of synapomorphies at internal nodes for the cladistic analysis mentioned in the text. (DOCX) [file pone.0046839.s001.docx]

# Supporting text (Text S1) to:

## A new rhynchocephalian from the Late Jurassic of Germany with a dentition that is unique amongst tetrapods

## Oliver W. M. Rauhut^1,2,^*, Alexander M. Heyng^2^, Adriana López-Arbarello^1^, and Andreas Hecker^3^

1Bayerische Staatssammlung für Paläontologie und Geologie and Department of Earth and Environmental Sciences, LMU Munich, Richard-Wagner-Straße 10, 80333 Munich, Germany

2GeoBioCenter, LMU Munich, Richard-Wagner-Straße 10, 80333 Munich, Germany

3Max-Planck-Gesellschaft, Generalverwaltung, Hofgartenstr. 8, 80343 Munich, Germany

*Author for correspondence (o.rauhut@lrz.uni-muenchen.de)

**1. Supplementary data**

**1. Characters used in the phylogenetic analysis**

Unless indicated otherwise, characters are taken from Apesteguia & Novas (2003) and Dupret (2004). See these papers for original sources of character definitions. Several characters have been modified, and a number of characters used by Apesteguía & Novas were found to be phylogenetically uninformative and have thus been removed.

1. Antorbital length / skull length ratio: More or equal than 1/3 (0); between 1/3 and ¼ (1); less or equal than ¼ (2) (Apesteguia & Novas, 2003).
2. Orbit diameter / skull length ratio: 1/3 or more (0); less than 1/3 (1) (Apesteguia & Novas, 2003).
3. Supratemporal fenestra / orbit length ratio: Less than 75% (0); more than 75% (1) (Apesteguia & Novas, 2003).
4. Supratemporal fenestra / skull length ratio: Less than ¼ (0); more than ¼ (1) (Apesteguia & Novas, 2003).
5. Lower temporal fenestra / skull length ratio: Less or equal than ¼ (0); more than ¼ (1) (Apesteguia & Novas, 2003).
6. Maxilla with premaxillary process: elongate (0); short or absent (1) (Apesteguia & Novas, 2003).
7. Premaxillary posterodorsal process (which excludes the maxilla from the external naris margin): absent (0); present (1) (Apesteguia & Novas, 2003).
8. Posterior end of the maxilla: narrow or tapers (0); broad dorso-ventrally (1) (Apesteguia & Novas, 2003).
9. Lacrimal: present (0); absent (1) (Apesteguia & Novas, 2003).
10. Jugal dorsal process: broad and short (0); narrow and elongate (1) (Apesteguia & Novas, 2003).
11. Posterior process of the jugal: absent or small (0); elongate (1) (modified from Dupret, 2004)
12. Prefrontal and postfrontal profusely sculptured: absent (0); present (1) (Apesteguia & Novas, 2003).
13. Prefrontal - jugal contact: absent (0); present (1) (Apesteguia & Novas, 2003).
14. Postorbital with marked dorsal ridge and deep ventrolateral concavity: absent (0); present (1) (Apesteguia & Novas, 2003).
15. Frontals: separate (0); fused (1) (Apesteguia & Novas, 2003).
16. Parietals: separate (0); fused (1) (Apesteguia & Novas, 2003).
17. Interparietal / interorbital width or intertemporal width of parietals: broader or greater than interorbital width (0); narrower or less than interorbital width (1) (Apesteguia & Novas, 2003).
18. Parietal crest: absent (0); present (1) (Apesteguia & Novas, 2003).
19. Posterior edge of parietals: greatly incurved (0); slightly incurved (1); convex (2) (Apesteguia & Novas, 2003).
20. Parietal foramen, position in respect to the anterior border of the supratemporal fenestra: posterior (0); at the same level or anterior (1) (Apesteguia & Novas, 2003).
21. Lower temporal bar shape / position: aligned with maxilary tooth row (0); bowed (1) (Apesteguia & Novas, 2003).
22. Lower temporal bar: incomplete (0); complete (1) (Apesteguia & Novas, 2003).
23. Palatines posterior shape: tapers posteriorly (0); relatively wide posteriorly (1) (Apesteguia & Novas, 2003).
24. Midline contact of the palatines: absent, palatines separated by pterygoids and vomer (0); present, no contact between pterygoid and vomer (1).
25. Pterygoid bones anterior contact: absent (0); slight (1); vast (2) (Apesteguia & Novas, 2003).
26. Interpterygoid vacuity posterior opening or relative separation of the pterygoid posteromedial processes: widely open (0); as wide as vacuity (1); almost closed (2) (Apesteguia & Novas, 2003).
27. Posterior border of ectopterygoid wing of pterygoid: approximately level with basicranial artriculation (0); offset anteriorly from basicranial articulation (1) (modified from Apesteguia & Novas, 2003: ch. 25).
28. Pterygoid: entering into margin of suborbital fenestra (0); excluded from margin of suborbital fenestra(1) (Apesteguia & Novas, 2003).
29. Parabasisphenoid depression: absent (0); present (1) (Dupret, 2004)
30. Q-Qj foramen size: small (0); enlarged (1) (Apesteguia & Novas, 2003).
31. Q-Qj foramen position: between Q and Qj (0); in Q (1) (Apesteguia & Novas, 2003).
32. Q-Qj conch size and emargination: pronounced (0); reduced (1) (Apesteguia & Novas, 2003).
33. Supratemporal: present (0); fused or absent (1) (Apesteguia & Novas, 2003).
34. Inferred jaw motion: orthal (0); propalineal (1) (Apesteguia & Novas, 2003).
35. Degree of propalinality, measured either as palatal tooth row extension or length in which palatines keep parallel to the maxillae: small palatal row, parallel line restricted to the anterior region (0); enlarged, palatines accompanying maxilla half its own length (1); palatines accompanying maxilla by its complete length, ‘eupropalinality’ (2) (Apesteguia & Novas, 2003).
36. Lower jaw, anterior region: toothed (0); edentate (1). (Apesteguia & Novas, 2003).
37. Ventral process at the anterior end of the dentary: absent (0); well-developed (1) (modified from Apesteguia & Novas, 2003).
38. Inclination of jaw symphysis in relation to long axis of the mandible (line from anterodorsal end of dentary to posterior end of glenoid): more than 50° (0); less than 50° (1); almost vertical ventrally and with pronounced anterior process dorsally (2) (modified from Apesteguia & Novas, 2003).
39. Jaw symphysis, dorsal development: moderately developed (0); well projected anterodorsally, comparable to a caniniform (1) (Apesteguia & Novas, 2003).
40. Mandibular foramen: small (0); large (1) (Apesteguia & Novas, 2003).
41. Glenoid cavity: smooth surface, lacking an anteroposterior central ridge (0); elongate and asymmetrical surface, with a strong anteroposterior central ridge (1); symmetrical facet with a strong anteroposterior central ridge (2) (Apesteguia & Novas, 2003).
42. Coronoid process: short, weak or absent, less than half jaw (0); pronounced, around half jaw (1); high, as tall as jaw (2) (Apesteguia & Novas, 2003).
43. Retroarticular process: pronounced (0); reduced, caudally projected (1), reduced, dorsally curved (2) (Apesteguia & Novas, 2003).
44. Posterior process of dentary: short, does not reach glenoid level (0); elongate, reaching glenoid level (1); elongate, reaching the end of glenoid level (2) (Apesteguia & Novas, 2003).
45. Marginal dental implantation: pleurodont (0); degree of posterior acrodonty (1); fully acrodont (2) (Apesteguia & Novas, 2003).
46. Tooth replacement: alternate (0); addition at back of jaw (1) (Apesteguia & Novas, 2003).
47. Dentary regionalization with small juvenile teeth (hatchling) in the anterior region of maxilla and dentary: absent, only pleurodont teeth (0); present, with hatchling pleurodont teeth (1); present, with hatchling, successional and additional acrodont teeth (2); absent, only additional acrodont teeth (3) (Apesteguia & Novas, 2003).
48. Anterior caniniform teeth in maxillae and dentaries: absent (0); present (1) (Apesteguia & Novas, 2003).
49. Lateral and medial wear facets on marginal teeth: absent or smooth (0); well established (1) (Apesteguia & Novas, 2003).
50. Marginal teeth breadth: equal to or lesser than length (0); square, expanded lateromedially (1); rectangular, wider than long (2) (Apesteguia & Novas, 2003) (ordered).
51. Number of premaxillary teeth: more than seven (0); seven to four (1); three or less (2) (Apesteguia & Novas, 2003).
52. Premaxillary teeth structure: discrete (0); chisel-like (1) (Apesteguia & Novas, 2003).
53. Posterior maxillary teeth: as large as (0) or smaller than more anterior teeth (1) (reversed from Dupret, 2004).
54. Maxillary teeth shape: simple cones (0); small occasional posteromedial flanges (1); extensive posteromedial flanges (2) (Apesteguia & Novas, 2003).
55. Maxillary teeth with anterolateral flange: absent (0); present (1) (Apesteguia & Novas, 2003).
56. Number of palatine tooth rows: more than one (0); a single row plus one isolated relictual tooth (1); a single lateral tooth row (2) (Apesteguia & Novas, 2003).
57. Lateral palatine tooth row: curves medially posteriorly (0); parallel to maxillary tooth row over ist entire length (1).
58. Palatine teeth with flanges: absent (0); present on some (1) (Apesteguia & Novas, 2003).
59. Number of pterygoid tooth rows: three or more rows (0); two rows (1); one row or absent (2) (Apesteguia & Novas, 2003).
60. Mandibular teeth with anterolateral flanges: absent (0); present, at least in one tooth (1) (Apesteguia & Novas, 2003).
61. Mandibular teeth with anteromedial flanges: absent (0); present (1) (Apesteguia & Novas, 2003).
62. Dental ridges in adult additional teeth: absent (0); present (1) (Apesteguia & Novas, 2003).
63. Number of presacral vertebrae: less than 25 (0); more than 25 (1) (Dupret 2004).
64. 2nd sacral vertebra with posterior process: small or absent (0); prominent (1) (modified from Apesteguia & Novas, 2003).
65. Coossification of scapula and coracoid: present (0); absent (1) (Dupret, 2004).
66. Ischium posterior border: posterior end uninterrupted (0); tubercle on posterior end (1); prominent posterior process (2) (Apesteguia & Novas, 2003).
67. Metatarsals I-IV longer than V (0); all metatarsals of subequal length (1) (Dupret, 2004).
68. Astragalus and calcaneum: unfused (0); fused (1) (Dupret, 2004).
69. Skull length: less than 30 mm (0); 30-90 mm (1); equal or larger than 100 mm (2) (Apesteguia & Novas, 2003).
70. Postzygapophyses, dorsal shape: flat (0); swollen (1) (Apesteguia & Novas, 2003).

**2. Data matrix**

The data matrix is based on Apesteguia & Novas (2003), with additions based on Dupret (2004) and own observations. The following taxa were added to the matrix, with bases for character codings noted in brackets: *Younginia* (outgroup; Gow 1975); *Gephyrosaurus* (Evans 1980); *Clevosaurus wangi* (Wu 1994; though see also Jones 2006 for the taxonomy of this taxon); *Oenosaurus* (BSPG 2009 I 23); *Planocephalosaurus* (Fraser 1982); *Pleurosaurus* (BSPG 1925 I 18, BSPG 1978 I 7, Carroll & Wild 1994, Dupret 2004). Symbols: R=0/1; S=1/2. A Nexus or MacClade version of the matrix is available from the corresponding author upon request.

________________________5____10____15____20____25____30____35_

*Younginia* 01000 00001 00000 00000 01100 001?0 1000?

Squamata 0RR0R 0?00R 00?00 00000 00000 0000? ?0?0?

*Gephyrosaurus* 00000 00000 000?1 10000 10001 00000 00100

*Brachyrhinodon* 21111 10111 100?0 00000 11001 10?0? ???0?

*Clevosaurus hudsoni* 11111 11111 10000 01000 11002 10100 00001

*Clevosaurus wangi* 11101 ?1111 ?0?00 ?0000 11002 111?0 100?1

*Cynosphenodon* ????? ????? ????? ????? ????? ??1?? ???1?

*Diphydontosaurus* 10000 00010 00001 00000 10001 00000 00101

*Eilenodon* ????? ????? ?1??? ????? ????? ????? ???1?

*Homoeosaurus* 0R000 00110 10010 000R1 11102 0010? ??10?

*Kallimodon* 01110 ?011? ?00?0 01111 11102 0111? ??101

*Oenosaurus* 20??? 0?1?? ?0?10 111?? ??012 2011? ???11

*Opisthias* ?1??? 11111 ??110 011?1 1?1?2 20??? ???12

*Palaeopleurosaurus* 01110 00010 00000 11110 00002 11011 10100

*Planocephalosaurus* 00000 00010 00001 10010 00001 00000 ?0100

*Pleurosaurus* 0110? 01010 00100 01111 00?12 11??? ?0100

*Pamizinsaurus* ????? ?1??? ?0??? ????? ????? 2?1?? ?0001

*Priosphenodon* 01100 11111 01110 01111 10102 201?0 00012

*Sapheosaurus* 11?10 0?1?0 ????0 01111 101?1 0011? ?????

*Sphenodon* 11111 00111 10010 01111 11102 21111 01112

*Toxolophosaurus* ????? ????? ????? ????? ????? ????? ???1?

*Zapatadon* 10?01 ??01? 0?1?? 1?1?1 ?1002 21011 0101?

_______________________40____45____50____55____60____65____70_

*Younginia* 00?00 ?0??0 00000 20100 00?0? ????0 2??10

Squamata 00000 00000 00000 00000 ???20 0?000 100?0

*Gephyrosaurus* 00000 ?0010 00000 00000 00000 0000? ?0???

*Brachyrhinodon* ?0001 ?1012 12010 10010 10?1? ??0?? ?0?0?

*Clevosaurus hudsoni* 11001 11012 12010 21020 10111 01010 10100

*Clevosaurus wangi* ????1 11?12 12010 ??020 10?11 ????? ?????

*Cynosphenodon* ?111? ?1??2 12110 21?00 ????1 0???? ???0?

*Diphydontosaurus* 00001 0101R 11000 10100 00000 ??01? 10?00

*Eilenodon* 1???1 20222 ?3012 2??21 2?1?1 11??? ???2?

*Homoeosaurus* ?1101 ?1112 12010 21020 0??21 ??010 20100

*Kallimodon* ?1001 ?2012 12010 21?20 21?21 01?10 20001

*Oenosaurus* 11101 12112 ??0?0 ??0?? 20?2? ????? ???0?

*Opisthias* 11011 ?0112 ?3011 2??2? 2?121 11?1? ???1?

*Palaeopleurosaurus* 111?1 ?10S2 12010 2102? 20?21 ??111 11010

*Planocephalosaurus* 01001 ?1??2 1?000 10010 0011? ?1??? ?????

*Pleurosaurus* 00101 11022 ??000 21101 21121 011?1 ?1110

*Pamizinsaurus* ?1101 ?11?2 120?0 2???? 2???1 ?1??? ???00

*Priosphenodon* 11211 20222 ?3012 21021 21121 11?1? ???21

*Sapheosaurus* 10001 ?20?? ??0?? 210?0 ????? ??011 20111

*Sphenodon* 11101 11112 12110 21010 21121 00000 10010

*Toxolophosaurus* 11211 ?0??2 ?3012 ????? ????1 1???? ???1?

*Zapatadon* ?111? ?1??2 120?0 ??01? 20?2? ????? ???0?

**3. Phylogenetic analysis**

The data matrix was analysed with PAUP 4.0b10 (Swofford 2003), using a branch and bound search. The initial analyses resulted in 119 trees with a length of 187 steps (CI 0.465, RI 0.649, RC 0.302). The strict consensus tree of these trees showed poor resolution, with *Oenosaurus* representing a member of a polytomy of rhynchocephalians more derived than *Planocephalosaurus* (Suppl. Fig. 1); only pleurosaurs and eilenodontines showed up as monophyletic clades within this polytomy. However, reduced consensus trees of these 119 trees recovered a monophyletic Sphenodontinae, including *Oenosaurus*, but without further resolution within this clade (Suppl. Fig. 2).

The taxon *Pamizinsaurus* from the Early Cretaceous of Mexico (Reynoso 1997) was identified as a wildcard taxon that strongly influenced the results. This may partially be due to high amounts of missing data (64 %) in this taxon; however, that several other taxa have even higher amounts of missing data (*Eilenodon* 68 %; *Cynosphenodon*: 73 %; *Toxolophosaurus*: 79 %) but proved to be less problematic, indicates that the wildcard status of *Pamizinsaurus* might also be due to some character conflict.

In a next step, we thus removed *Pamizinsaurus* from the matrix and ran a second analysis with the same settings as above. The analysis resulted in only two most parsimonious trees with a length of 184 steps (CI 0.473, RI 0.654, RC 0.309). The strict consensus tree of these two trees (Fig. 3 and Suppl Fig. 3) is considerably better resolved than that resulting from the full analysis, but, importantly, it does not contradict the results of the latter, neither in the strict consensus, nor in any reduced consensus tree. Thus, we chose these results to illustrate and further explore the relationships of *Oenosaurus*. Bootstrap (10000 replicates) and Bremer support analyses were carried out on the pruned matrix, and the following list of synapomorphies is based on the strict consensus tree resulting from the pruned analysis.


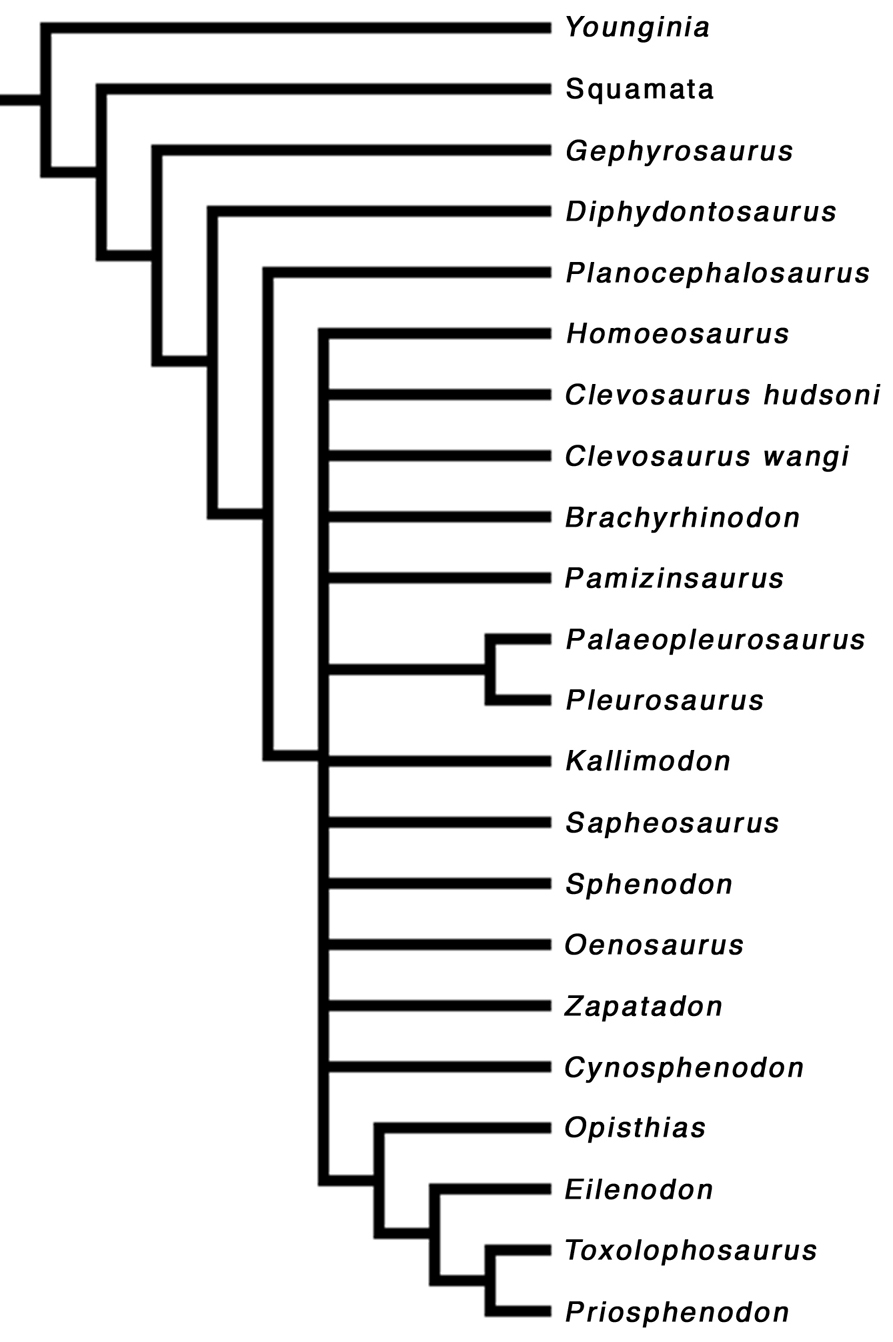


Suppl. Fig. 1: Strict consensus tree of 119 equally parsimonious trees resulting from the full analysis including *Pamizinsaurus*. See text for details.


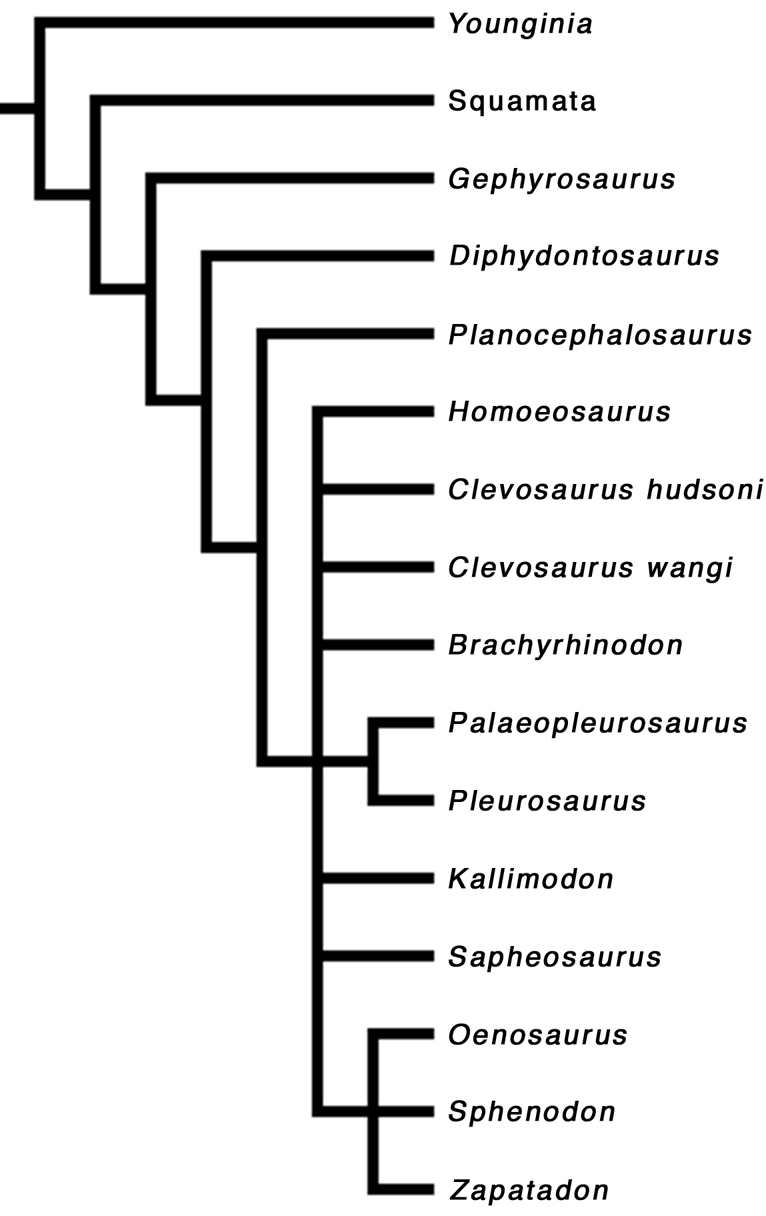


Suppl. Fig. 2: Reduced consensus tree of 119 trees resulting from the full analysis. In this tree, *Oenosaurus* is shown to be more closely related to sphenodontines than to other sphenodontids (with the exception of opisthodontians, which were removed from this reduced consensus tree).


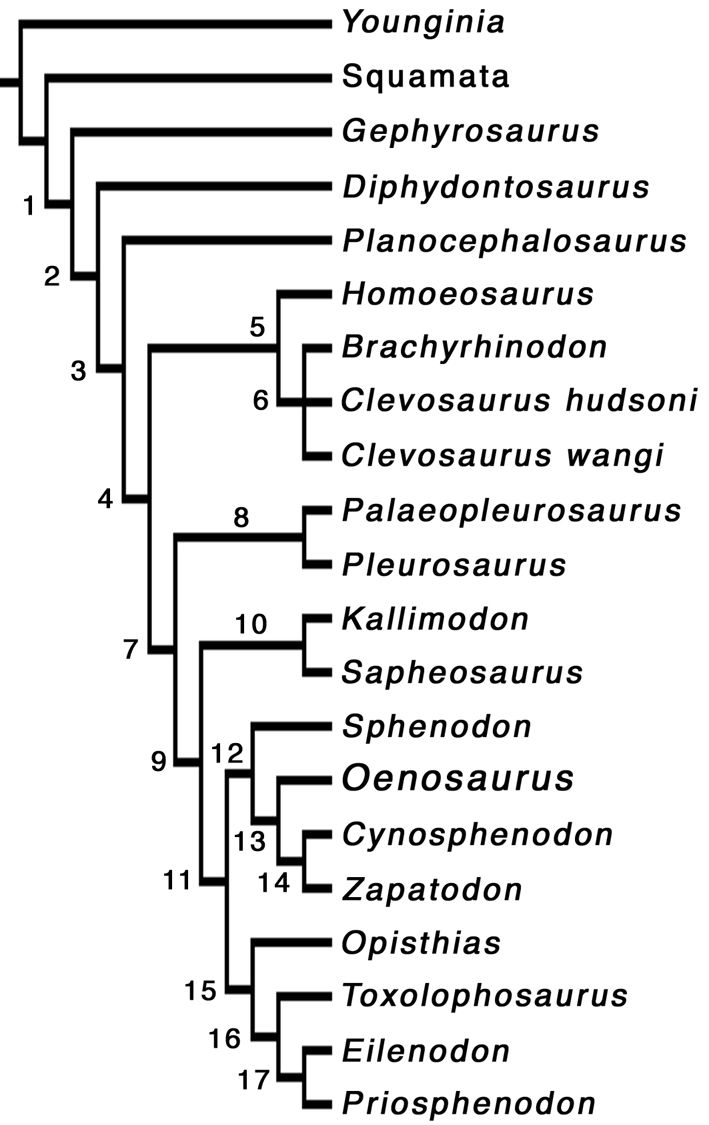


Suppl. Fig. 3: Strict consensus tree of 2 trees resulting from the pruned data matrix, excluding *Pamizinsaurus* (see Fig. 3), with internal nodes labelled.

**4. List of synapomorphies**

The following list of synapomorphies of Rhynchocephalia and more inclusive clades is based on the strict consensus tree of two most parsimonious trees resulting from the pruned analysis (Suppl Fig. 3). Synapomorphies were evaluated using the trace character option in MacClade 4.08 (Maddison & Maddison 2003). Synapomorphies present in *Oenosaurus* are indicated by an asterisk (*). Numbers in brackets next to node numbers and/or names indicate Bootstrap (in %) and Bremer support of the respective nodes. Numbers before character description refer to characters in character list. Node names follow Reynoso (1996) and Apesteguia & Novas (2003).

**Node 1: Rhynchocephalia (77%; 3)**

Unambiguous synapomorphies: 15: Frontals fused (reversed in sphenodontids); 21: Lower temporal bar bowed laterally (reversed in *Planocephalosaurus* and pleurosaurids); 25: Small anterior contact of the pterygoids present*; 44: Posterior process of the dentary reaches the level of the glenoid*.

**Node 2: Sphenodontia (78%; 4)**

Unambiguous synapomorphies: 9: Lacrimal absent; 40: Enlarged mandibular foramen*; 42: Pronounced coronoid process present* (reversed in opisthodontians); 46: New teeth added at back of jaw; 47: Dentary regionalization with small juvenile teeth anteriorly; 51: Seven or less premaxillary teeth; 64: Prominent posterior process on the second sacral vertebra (reversed in *Sphenodon* and unknown in other sphenodontines).

**Node 3 (74%; 2)**

Unambiguous synapomorphies: 37: Well-developed ventral process at the anterior end of the dentary present*; 45: Acrodont tooth implantation*; 54: Maxillary teeth with posteromedial flanges; 58: Palatine teeth with flanges; 59: Less than three rows of pterygoid teeth*.

Possible synapomorphies under accelerated transformation: 19: Posterior margin of parietals only slightly curved (absent in clevosaurs); 41: Glenoid cavity elongate, with a strong anteroposterior central ridge* (unknown in *Planocephalosaurus*); 60: Anterolateral flanges in mandibular teeth (unknown in *Planocephalosaurus*).

**Node 4: Sphenodontidae (94%; 6)**

Unambiguous synapomorphies: 2: Orbit diameter less than 1/3 of skull length (reversed at node 13); 15: Frontals unfused* (reversal of a rhynchocephalian synapomorphy); 25: Anterior contact of the pterygoids extensive*; 36: Anterior region of lower jaw edentate*; 49: Well-developed lateral and medial wear facets on marginal teeth; 51: Three or less premaxillary teeth; 52: Premaxillary teeth chisel-like (reversed in *Brachyrhinodon*).

Possible synapomorphies under accelerated transformation: 3: Supratemporal fenestra more than 75% of the length of the orbit (not present in *Homeosaurus*); 8: Posterior end of the maxilla broad dorso-ventrally* (absent in pleurosaurids); 20: Parietal foramen at the same level as or anterior to the anterior margin of the supratemporal fenestrae (absent in clevosaurus and *Palaeopleurosaurus*); 28: Pterygoid excluded from the margin of the infratemporal fenestra* (absent in *Palaeopleurosaurus* and unknown in *Pleurosaurus*); 59: Only one or no row of pterygoid teeth* (present in *Homeosaurus*, but absent in clevosaurs).

Possible synapomorphies under delayed transformation: 41: Glenoid cavity elongate, with a strong anteroposterior central ridge* (unknown in *Planocephalosaurus*); 60: Anterolateral flanges in mandibular teeth (unknown in *Planocephalosaurus*).

**Node 5 (<50%; 1)**

Unambiguous synapomorphies: 11: Posterior process of the jugal elongate (convergently present in *Sphenodon*); 22: Lower temporal bar complete (convergently present in *Kallimodon* and sphenodontines); 68: Fused astragalus and calcaneum (convergently present in *Pleurosaurus* and *Sapheosaurus*).

Possible synapomorphies under delayed transformation: 8: Posterior end of the maxilla broad dorso-ventrally (also present in other sphenodontids, with the exception of pleurosaurids).

**Node 6: Clevosaurs (70%; 2)**

Unambiguous synapomorphies: 1: Antorbital skull length less than 1/3 of the total skull length (convergently present in *Diphydontosaurus*, *Sapheosaurus* and sphenodontines); 5: Length of the lower temporal fenestra more than 1/4 of skull length (convergently present in sphenodontines); 6: Premaxillary process of the maxilla short or absent (convergently present in opisthodontians); 10: Jugal dorsal process narrow and elongate (convergently present at node 11); 26: Separation of the pterygoid posteromedial processes as wide as interpterygoid vacuity (convergently present in pleurosaurids); 56: Single palatine tooth row plus one isolated relictual tooth present.

Possible synapomorphies under delayed transformation: 3: Supratemporal fenestra more than 75% of the length of the orbit (also present in other sphenodontids, with the exception of *Homeosaurus*).

**Node 7 (<50%; 1)**

Unambiguous synapomorphies: 17: Intertemporal width of parietals less than interorbital width* (convergently present in *Clevosaurus hudsoni*); 18: Parietal crest present*; 29: Parabasisphenoid depression present*; 56: Single lateral tooth row on the palatine*.

Possible synapomorphies under accelerated transformation: 30: Enlarged quadrate foramen present (unknown in most taxa at this node, but present in *Palaeopleurosaurus* and sphenodontines, but absent in *Priosphenodon*); 57: Lateral palatine tooth row parallel to maxillary tooth row over its entire length (equivocal in pleurosaurids and reversed at node 13).

Possible synapomorphies under delayed transformation: 3: Supratemporal fenestra more than 75% of the length of the orbit (also present in clevosaurs, but not in *Homeosaurus*); 19: Posterior margin of parietals only slightly curved (also present in *Planocephalosaurus*, but absent in clevosaurs); 59: Only one or no row of pterygoid teeth* (present in *Homeosaurus*, but absent in clevosaurs).

**Node 8: Pleurosauridae (60%; 2)**

Unambiguous synapomorphies: 21: Lower temporal bar aligned with maxillary tooth row (reversal of a rhynchocephalian synapomorphy); 26: Separation of the pterygoid posteromedial processes as wide as interpterygoid vacuity (convergently present in clevosaurs); 38: Inclination of jaw symphysis less than 50° (convergently present in *Homeosaurus* and sphenodontines); 63: More than 25 presacral vertebrae; 65: Scapula and coracoid not coossified (convergently present in *Sapheosaurus*); 67: All metatarsals of subequal length.

**Node 9 (<50%; 1)**

Unambiguous synapomorphies: 23: Palatines wide posteriorly (convergently present in *Homeosaurus* and reversed at node 13).

Possible synapomorphies under accelerated transformation: 14: Postorbital with marked dorsal ridge and deep ventrolateral concavity* (unknown in *Kallimodon* und *Sapheosaurus* and convergently present in *Homeosaurus*); 70: Postzygapophyses dorsally swollen (absent in *Sphenodon* and unknown in most other higher sphenodontids).

Possible synapomorphies under delayed transformation: 8: Posterior end of the maxilla broad dorso-ventrally* (also present in clevosaurs and *Homeosaurus*); 20: Parietal foramen at the same level as or anterior to the anterior margin of the supratemporal fenestrae (also present in *Homeosaurus* and *Pleurosaurus*, but absent in clevosaurus and *Palaeopleurosaurus*); 28: Pterygoid excluded from the margin of the infratemporal fenestra* (also present at node 5, but absent in *Palaeopleurosaurus* and unknown in *Pleurosaurus*); 57: Lateral palatine tooth row parallel to maxillary tooth row over its entire length (equivocal in pleurosaurids and reversed at node 13).

**Node 10 (<50%; 1)**

Unambiguous synapomorphies: 4: Supratemporal fenestra more than 1/4 of the skull length (convergently present in *Brachyrhinodon*, *Clevosaurus hudsoni*, *Palaeopleurosaurus* and *Sphenodon*); 42: Coronoid process very high, as high as jaw (convergently present in *Oenosaurus*); 66: Prominent posterior process on the ischium (convergently present in *Homeosaurus*).

Possible synapomorphies under delayed transformation: 70: Postzygapophyses dorsally swollen (also present in *Priosphenodon*, but absent in *Sphenodon* and unknown in all other higher sphenodontids).

**Node 11 (<50%; 1)**

Unambiguous synapomorphies: 10: Jugal dorsal process narrow and elongate (convergently present in clevosaurs); 26: Interpterygoid vacuity almost closed or closed posteriorly*; 34: Propalineal jaw motion*; 43: retroarticular process reduced and posteriorly directed* (convergently present in *Homeosaurus*)

Possible synapomorphies under accelerated transformation: 13: Prefrontal-jugal contact present (ambiguous within sphenodontines and convergently present in *Pleurosaurus*).

**Node 12: Sphenodontinae (<50%; 2)**

Unambiguous synapomorphies: 1: Antorbital skull length less than 1/3 of the total skull length* (convergently present in *Diphydontosaurus*, *Sapheosaurus* and clevosaurs); 5: Length of the lower temporal fenestra more than 1/4 of skull length (convergently present in clevosaurs); 22: Lower temporal bar complete (convergently present at node 5 and in *Kallimodon*); 32: Q-Qj conch size and emargination reduced; 38: Inclination of jaw symphysis less than 50°* (convergently present in *Homeosaurus* and pleurosaurids).

Possible synapomorphies under delayed transformation: 30: Enlarged quadrate foramen present (also present in *Palaeopleurosaurus*, but unknown in most derived sphenodontids and absent in *Priosphenodon*).

**Node 13 (<50%; 1)**

Unambiguous synapomorphies: 2: Orbit diameter more than 1/3 of skull length*; 16: Parietals fused* (convergently present in *Gephyrosaurus*, *Planocephalosaurus* and *Palaeopleurosaurus*); 23: Palatines narrow posteriorly* (reversal of a synapomorphy at node 9); 57: Palatine tooth row curves medially posteriorly* (reversal of a synapomorphy at node 7 or 9).

**Node 14 (<50%; 1)**

Unambiguous synapomorphies: 39: Jaw symphysis projected anterodorsally (convergently present in opisthodontians).

**Node 15: Opisthodontia (98%; 5)**

Unambiguous synapomorphies: 6: Premaxillary process of the maxilla short or absent (convergently present in clevosaurs); 7: Posterodorsal process of the premaxilla present (convergently present in *Clevosaurus* and *Pleurosaurus*); 39: Jaw symphysis projected anterodorsally (convergently present at node 14); 47: Dentary without retained anterior juvenile teeth, only additional posterior teeth present; 50: Marginal teeth expanded transversely; 61: Mandibular teeth with anteromedial flanges.

Possible synapomorphies under accelerated transformation: 12: Prefrontal and postfrontal sculptured (unknown in *Opisthias*); 41: Symmetrical articular facet with pronounced central ridge in the mandibular glenoid (unknown in *Opisthias*); 55: Maxillary teeth with anterolateral flange (unknown in *Opisthias* and convergently present in *Pleurosaurus*).

Possible synapomorphies under delayed transformation: 13: Prefrontal-jugal contact present (ambiguous within sphenodontines and convergently present in *Pleurosaurus*).

**Node 16: Eilenodontinae (86%; 2)**

Unambiguous synapomorphies: 43: Retroarticular process reduced and dorsally curved.

Possible synapomorphies under accelerated transformation: 38: Jaw symphysis almost vertical and with pronounced ventral process (unknown in *Eilenodon*); 44: Posterior process of the dentary reaches the level of the posterior end of the glenoid (convergently present in *Pleurosaurus*); 50: Marginal teeth rectangular, wider than long.

Possible synapomorphies under delayed transformation: 12: Prefrontal and postfrontal sculptured (unknown in *Opisthias*); 41: Symmetrical articular facet with pronounced central ridge in the mandibular glenoid (unknown in *Opisthias*); 55: Maxillary teeth with anterolateral flange (unknown in *Opisthias* and convergently present in *Pleurosaurus*).

**Node 17 (77%; 1)**

Possible synapomorphies under delayed transformation: 38: Jaw symphysis almost vertical and with pronounced ventral process (unknown in *Eilenodon*).

**References:**

Apesteguia, S., and F. E. Novas. 2003. Large Cretaceous sphenodontian from Patagonia provides insight into lepidosaur evolution in Gondwana. Nature 425:609-612.

Carroll, R. L., and R. Wild. 1994. Marine members of the Sphenodontia. Pp. 70-83. *In* N. C. Fraser, and H.-D. Sues, eds. In the shadow of the dinosaurs. Early Mesozoic tetrapods. Cambridge University Press, Cambridge.

Dupret, V. 2004. The pleurosaurs: anatomy and phylogeny. Revue de Paléobiologie, Vol. spéc. 9:61-80.

Evans, S. E. 1980. The skull of a new eosuchian reptile from the Lower Jurassic of South Wales. Zoological Journal of the Linnean Society 70:203-264.

Fraser, N. C. 1982. A new rhynchocephalian from the British Upper Trias. Palaeontology 25(4):709-725.

Gow, C. E. 1975. The morphology and relationships of *Youngina capensis* Broom and *Prolacerta broomi* Parrington. Palaeontologia africana 18:89-131.

Jones, M. E. H. 2006. The Early Jurassic clevosaurs from China (Diapsida: Lepidosauria). New Mexico Museum of Natural History & Science Bulletin 37:548-562.

Maddison, W. P., and D. R. Maddison. 2003. MacClade, version 4.06. Sinauer Associates Inc., Sunderland, Massachussets.

Reynoso, V.-H. 1996. A Middle Jurassic *Sphenodon*-like sphenodontian (Diapsida: Lepidosauria) from Huizachal Canyon, Tamaulipas, Mexico. Journal of Vertebrate Paleontology 16(2):210-221.

Reynoso, V.-H. 1997. A "beaded" sphenodontian (Diapsida: Lepidosauria) from the Early Cretaceous of central Mexico. Journal of Vertebrate Paleontology 17(1):52-59.

Swofford, D. L. 2003. PAUP*. Phylogenetic Analysis Using Parsimony (*and other methods). Version 4. Sinauer Associates, Sunderland, Massachusetts.

Wu, X.-C. 1994. Late Triassic - Early Jurassic sphenodontians from China and the phylogeny of the Sphenodontia. Pp. 38-69. *In* N. C. Fraser, and H.-D. Sues, eds. In the shadow of the dinosaurs. Early Mesozoic tetrapods. Cambridge University Press, Cambridge.
